# Effectiveness of NLRP3 Inhibitor as a Non-Hormonal Treatment for ovarian endometriosis

**DOI:** 10.1186/s12958-022-00924-3

**Published:** 2022-03-29

**Authors:** Mayuko Murakami, Satoko Osuka, Ayako Muraoka, Shotaro Hayashi, Bayasula, Yukiyo Kasahara, Reina Sonehara, Yumi Hariyama, Kanako Shinjo, Hideaki Tanaka, Natsuki Miyake, Sayako Yoshita, Natsuki Nakanishi, Tomoko Nakamura, Maki Goto, Hiroaki Kajiyama

**Affiliations:** 1 Department of Obstetrics and Gynecology, Nagoya University Graduate School of Medicine, 65 Tsurumai-cho, Showa-ku, Nagoya, Aichi 466-8550 Japan; 2 Bell Research Center for Reproductive Health and Cancer, Nagoya University Graduate School of Medicine, Nagoya, Aichi Japan; 3 Department of Obstetrics and Gynecology, Toyota Kosei Hospital, Toyota, Aichi Japan

**Keywords:** Endometriosis, Infertility, NLRP3 inflammasome, MCC950, Non-hormonal therapies, IL-1β, Murine model

## Abstract

**Background:**

Endometriosis is a complex syndrome characterized by an estrogen-dependent chronic inflammatory process that affects 10% of women of reproductive age. Ovarian endometriosis (OE) is the most common lesion in endometriosis and may cause infertility, in addition to dysmenorrhea. Hormonal treatments, which are the conventional treatment methods for endometriosis, suppress ovulation and hence are not compatible with fertility. The inflammasome is a complex that includes Nod-like receptor (NLR) family proteins, which sense pathogen-associated molecular patterns and homeostasis-altering molecular processes. It has been reported that the nucleotide-binding oligomerization domain, leucine-rich repeat, and pyrin domain-containing (NLRP) 3 inflammasome, which contributes to the activation of interleukin-1 beta (IL-1β), might be related to the progression of endometriosis. Therefore, the aim of the present study was to evaluate non-hormonal therapies for OE, such as inhibitors of the NLRP3 inflammasome.

**Methods:**

The expression of NLRP3 was measured in the eutopic endometrium (EM) of patients with and without endometriosis and OE samples, as well as stromal cells derived from the endometrium of patients with and without endometriosis and OE samples (endometrial stromal cells with endometriosis [ESCs] and cyst-derived stromal cells [CSCs]). The effects of an NLRP3 inhibitor (MCC950) on ESCs and CSCs survival and IL-1β production were evaluated. We then administered MCC950 to a murine model of OE to evaluate its effects on OE lesions and ovarian function.

**Results:**

NLRP3 gene and protein expression levels were higher in OE and CSCs than in EM and ESCs, respectively. MCC950 treatment significantly reduced the survival of CSCs, but not that of ESCs. Moreover, MCC950 treatment reduced the co-localization of NLRP3 and IL-1β in CSCs, as well as IL-1β concentrations in CSCs supernatants. In the murine model, MCC950 treatment reduced OE lesion size compared to phosphate-buffered saline treatment (89 ± 15 *vs.* 49 ± 9.3 mm^3^ per ovary; *P* < 0.05). In the MCC950-treated group, IL-1β and Ki67 levels in the OE-associated epithelia were reduced along with the oxidative stress markers of granulosa cells.

**Conclusions:**

These results indicated that NLRP3/IL-1β is involved in the pathogenesis of endometriosis and that NLRP3 inhibitors may be useful for suppressing OE and improving the function of ovaries with endometriosis.

**Supplementary Information:**

The online version contains supplementary material available at 10.1186/s12958-022-00924-3.

## Background

Endometriosis is a complex syndrome characterized by an estrogen-dependent chronic inflammatory process that affects 10% of women of reproductive age [1, 2]. Ovarian endometriosis (OE) is the most common lesion in endometriosis [3] and causes infertility, in addition to dysmenorrhea [4]. Currently, the most common treatments for endometriosis are surgery and pharmacotherapy, including hormone therapy and non-steroidal anti-inflammatory drugs for pain relief. Oral contraceptives/low-dose estrogen and progestin, dienogest, levonorgestrel-releasing intrauterine system, and gonadotropin-releasing hormone (GnRH) analogs have been shown to be equally effective in improving endometriotic pain. GnRH antagonists should be used with caution for ovarian hormone deficiency symptoms and bone loss due to low estrogen status [5, 6]. Although surgical excision of endometriosis improves pain and enhances fertility [7, 8], a recent systematic review of the literature estimated the recurrence rate of endometriosis to be 21.5% at 2 years and 40%–50% at 5 years [9], and that recurrence and repeated surgery can further exacerbate pain and reduce fertility [10]. Therefore, regular and prolonged medication is highly recommended to prevent postoperative recurrence of endometriosis [11]. However, because estrogen is involved in the development of endometriosis, these hormone therapies suppress follicular development and ovulation. Therefore, the treatment of endometriosis in women who wish to become pregnant is very difficult, and non-hormonal drugs that can prevent the progression of endometriosis and contribute to ameliorating the biochemical mechanisms responsible for endometriotic infertility (such as oxidative stress on follicles due to OE) are desirable [5, 6, 12].

The development of endometriosis involves interaction of the endocrinal, immunological, pro-inflammatory, and pro-angiogenic processes [12]. Sampson’s theory has been accepted as a strong hypothesis [13], but there must be other factors that determine the ability of endometrial cells to adhere to peritoneal or ovarian surfaces, proliferate, and develop into endometriotic lesions, because retrograde menstruation is common in all women of reproductive age [14, 15]. Chronic inflammation is one of the distinguishing features of endometriosis, and a relationship between cell adhesion and inflammation has been reported [16, 17].

The inflammasome is a complex that can contain Nod-like receptor (NLR) family proteins, leucine-rich repeat, and pyrin domain-containing (NLRP) 1b, NLRP3, NLRP6, NLRP9b, or NLR family caspase recruitment domain (CARD)-containing protein (NLRC) 4, which senses pathogen-associated molecular patterns, danger-associated molecular patterns (DAMPs), and homeostasis-altering molecular processes [18, 19]. Previous studies have reported that the NLRP3 inflammasome may be related to the progression of endometriosis [14, 20].

NLRP3 is involved in hereditary Cryopyrin-associated periodic syndromes, such as Muckle–Wells syndrome, diabetic retinopathy, colorectal inflammatory disease, and gouty arthritis; treatment with NLRP3 inhibitors has been reported in these diseases [21–24]. Our group has focused our attention on MCC950, a small-molecule compound with high specificity for NLRP3 [21]. We hypothesized that inhibiting NLRP3 with MCC950 would suppress interleukin-1 beta (IL-1β) and alleviate endometriosis.

In this study, we first examined the expression of NLRP3 in the eutopic endometrium (EM) with and without endometriosis and OE samples. We confirmed that MCC950 treatment inhibits the secretion of IL-1β in primary human endometrial stromal cells. Finally, we examined the effect of MCC950 on endometriotic lesions in a murine model of OE that has been established previously [25].

## Materials and methods

### Patients and sample collection

Patients diagnosed with endometriomas, uterine leiomyomas, carcinoma in situ (CIS), and early-stage cervical cancer, who were referred to the Nagoya University Hospital and Toyota Kosei Hospital between July 2018 and November 2021, were enrolled in this study. The ethics committee of the Nagoya University Graduate School of Medicine (2014–0134) and Toyota Kosei Hospital (2018-ST01) approved the experiments. Written informed consent was obtained from each patient prior to participation in the study.

OE samples and endometrial tissues resected for therapeutic purposes from patients with endometriosis (eEM), were collected from 25 patients with OE and 20 patients with eEM and used for quantitative reverse transcription polymerase chain reaction (qRT-PCR), western immunoblotting, and stromal cell isolation, depending on the sample volume. Paraffin-embedded tissues of OE and endometrial samples that had been resected for therapeutic purposes were used for immunohistochemical analyses. Normal endometrial tissues (nEM) were obtained and evaluated from 10 patients with uterine leiomyomas, CIS, and stage IB1 cervical cancer without endometriosis and from an additional 6 patients for cell isolation.

### Primary human ESC and CSC isolation

Primary human endometrial stromal cells (obtained from patient with endometriosis [ESCs], without endometriosis [ESCsn]) and endometriotic cyst-derived stromal cells (CSCs) were isolated from human endometrial biopsies or resected endometriomas. Tissue biopsies were finely chopped in Dulbecco’s modified Eagle’s medium (DMEM; Nacalai Tesque, Kyoto, Japan). Chopped tissues were incubated with collagenase solution (1 mg/mL; FUJIFILM Wako Pure Chemical Corporation, Osaka, Japan) for 30 min at 37 °C, and the cell suspension was filtered through 70 μm filter membranes, followed by centrifugation to obtain a stromal cell pellet. ESCs/ESCsn and CSCs were then resuspended in fresh DMEM containing 10% fetal bovine serum (Cosmo Bio, Tokyo, Japan), 100 IU/mL penicillin, 100 mg/L streptomycin, and 25 mg/L amphotericin B. The next day, media containing unattached cells were transferred to a second dish before the media was removed and discarded. The cells were routinely maintained at 37 °C until they reached 90% confluence and were then seeded for experimental purposes, as detailed below. In total, ESCsn from 6 patients, ESCs from 15 patients, and CSCs from 15 patients were isolated, and cells were used for sequential experiments at 2–10 passages, depending on their growth status.

### Real-time quantitative polymerase chain reaction

Gene expression was analyzed using SYBR™ Green-based qRT-PCR of cDNA synthesized from sample-extracted mRNA. Tissues or ESCs/CSCs were washed with phosphate-buffered saline (PBS), and total RNA was isolated using the RNeasy™ Mini Kit (Qiagen, Hilden, Germany). RNA was measured using a NanoDrop™ ND1000 spectrophotometer (NanoDrop™ Technologies, USA). Next, 2 μg of total RNA from each sample was used for reverse transcription with 5 × RT Master Mix (Toyobo, Osaka, Japan), to generate first-strand cDNA in a 20 μL reaction mixture. The cDNA was diluted at a ratio of 1:10, and qRT-PCR was performed in a 96-well with 0.2 mL thin-walled PCR tubes using the LightCycler® 96 system (Roche, Basel, Switzerland). The real-time PCR mixture contained KOD SYBR® qPCR Mix (10 μL; Toyobo), primers (2 μM), and cDNA template (2 μg) in a total volume of 20 μL. Quantitative RT-PCR was performed to measure mRNA expression with the following primers: human *NLRP3* (forward, 5'-GCACCTGTTGTGCAATCTGAA-3'; reverse, 5'-TCCTGACAACATGCTGATGTGA-3'), human *NLRP1* (forward, 5'-CCACAACCCTCTGTCTACATTAC-3'; reverse, 5'-GCCCCATCTAACCCATGCTTC-3'), human *NLRC4* (forward, 5'-GGAAAGTGCAAGGCTCTGAC-3'; reverse, 5'-TGTCTGCTTCCTGATTGTGC-3'), human absent in melanoma 2 (*AIM2*) (forward, 5'-CTGCAGTGATGAAGACCATTCGTA-3'; reverse, 5'-GGTGCAGCACGTTGCTTTG-3'), and glyceraldehyde-3-phosphate dehydrogenase (*GAPDH*) (forward, 5'-CAGCCTCAAGATCATCAGCA-3'; reverse, 5'-GTCTTCTGGGTGGCAGTGAT-3'). The PCR conditions used were as follows: initial incubation at 98 °C for 2 min, denaturation at 98 °C for 10 s, annealing at 60 °C for 10 s (55 cycles), and extension at 68 °C for 30 s. Quantitative RT-PCR was performed in triplicate for all samples. Quantification was performed by calculating the ratio of the expression of gene of interest to that of *GAPDH* using the comparative C_t_ method.

### Western immunoblotting

Tissues or cells were lysed using RIPA lysis buffer (10 × ; Millipore, Burlington, MA, USA) containing 0.5 M Tris–HCl (pH 7.4), 1.5 M NaCl, 2.5% deoxycholic acid, 10% NP-40, and 10 mM EDTA in Milli-Q H_2_O (Roche). Lysates were clarified by means of centrifugation at 8000 × *g* for 10 min at 4 °C, following which the supernatants were collected.

Protein concentration was quantified using a BCA protein assay kit (Thermo Fisher Scientific, Waltham, MA, USA). Proteins were separated using 12.5% SDS-PAGE and transferred to a polyvinylidene difluoride membrane. The membrane was blocked for 1 h at room temperature with 5% (v/v) non-fat dry milk. After three washes with PBS containing Tween (PBST), the membrane was incubated in PBS at 4 °C, overnight, with anti-NLRP3 (19,771–1-AP, 1:1000, AdipoGen Life Sciences, Liestal, Switzerland), anti-IL-1β (No.12242, 1:100, Cell Signaling Technology, Danvers, MA, USA), and anti-caspase1 (#2225, 1:1000, Cell Signaling Technology). The membrane was again washed with PBST and incubated for 1 h at room temperature with a horseradish peroxidase-conjugated secondary antibody. Signals were developed using a standard ECL western blot detection reagent (Amersham Biosciences, Arlington Heights, IL, USA). Densitometric analysis was performed using ImageJ software version 2.2.0 (https://imagej.net/).

### Cell viability assay

The effect of MCC950 on cell viability was determined using cell counting. ESCs/CSCs (1.5 × 10^5^ cells) were seeded into each well of a 6-well plate and allowed to adhere overnight. The cells were cultured in serum-free media (SFM) for 24 h, to starve them, following which they were treated with different concentrations of MCC950 (0.1, 1, 10, and 100 μM; AG-CR1-3615-M005, AdipoGen Life Sciences) for 24 h. Finally, 1 mL of 0.25% trypsin was added to each well, and the cells were incubated with it for 5 min at 37 °C, after which the cells were collected, centrifuged, and the number of viable cells was counted.

### Immunocytochemistry

ESCs and CSCs were cultured on coverslips, and the medium was replaced with SFM containing MCC950 (100 μM). After incubation for 16 h, the cells were fixed in methanol for 2 min at room temperature and permeabilized with 0.5% Triton X-100 for 1 min. After blocking with 1% bovine serum albumin for 1 h at room temperature, the cells were incubated with primary antibodies against NLRP3 [19771–1-AP, 1:200, Proteintech, Chicago, IL, USA], IL-1β [sc-32294, 1:100, Santa Cruz Biotechnology, Heidelberg, Germany], CD10 [sc-9149, 1:50, Santa Cruz Biotechnology], vimentin [sc-6260, 1:50, Santa Cruz Biotechnology], and fibronectin [ab2413, 1:200, Abcam, Cambridge, UK] for 2 h at room temperature. The cells were then incubated with goat anti-rabbit (Alexa Fluor® 568, 1:500, Thermo Fisher Scientific, for anti-NLRP3 antibody) and goat anti-mouse (Alexa Fluor® 488, 1:500, Thermo Fisher Scientific, for anti-IL-1β antibody) secondary antibodies for 1 h at room temperature. Nuclear staining was carried out using 4',6-diamidino-2-phenylindole (4083, 1:1000, Cell Signaling Technology). Visualization was performed using a confocal laser-scanning microscope (BZ9000, Keyence, Osaka, Japan).

### Enzyme-linked immunosorbent assay (ELISA)

ESCs/CSCs were seeded at a density of 1 × 10^6^ cells/well in 6-well plates. The following day, the medium was replaced with SFM containing MCC950 (100 μM). After incubation for 16 h, the cell culture supernatants were collected. ELISAs were conducted on the culture media collected after treatment. Media samples were immediately centrifuged for 5 min at 8000 × *g* to collect the conditioned culture supernatant, which was stored at –80 °C until use. IL-1β and IL-18 released by ESCs and CSCs were measured using ELISA kits for IL-1β (DuoSet, R&D Systems, Minneapolis, MN, USA) and IL-18 (ab215539, Abcam), according to the manufacturer’s protocol.

### Animal model of endometriosis and MCC950 treatment

All animal experiments were approved by the Animal Experimental Committee of the Nagoya University Graduate School of Medicine (31452). We used a murine OE model, as described by Hayashi (2020) [25]. C57BL/6 N female mice (8 weeks of age) were purchased from Japan SLC (Shizuoka, Japan). Before starting the experiments, the animals were acclimatized for 7 d in an environment maintained at 23–25 °C with a 12 h/12 h dark/light cycle and given standard chow (CE-2; CLEA Japan, Tokyo, Japan) and water in a pathogen-free environment. The cages were changed weekly.

Donor female mice (9 weeks of age; n = 16) were euthanized to obtain the uterine tissue, which was cleaned of supplementary fibroadipose tissues using PBS. The uterus was cut longitudinally with a linear incision and minced (approximately 0.5 mm in diameter) with scissors. Following that, it was incubated with collagenase solution (1 mg/mL; FUJIFILM Wako Pure Chemical Corporation) and centrifuged to remove the supernatant containing collagenase. The pellet of the uterine tissue was immediately used for transplantation. Sixteen female mice were used as the recipients of uterine pellets for OE. The mice underwent uterine transplantation after a week of acclimatization. Induction and maintenance of systemic anesthesia were achieved with isoflurane (3% for induction and 2.5% for maintenance). Incisions of 5–7 mm were performed on the bilateral back skin and muscle layers, to search for the ovaries. Half of the uterine tissue pellet prepared from one donor female mouse was placed equally over each surface of the bilateral ovaries. Ovaries with attached uterine pellets were then pushed back into the peritoneal cavity, following which the incisions were closed.

Over the next four weeks, the recipient mice were treated with a single intraperitoneal injection of MCC950 (*n* = 8; 20 mg/kg) or PBS (*n* = 8), three times a week. The first injection of MCC950 was administered 1 h before inoculation of the donor’s uterine tissues.

### Histological analysis and measurement of murine endometriotic cysts

The endometriotic cysts were excised at 13 weeks of age as a single mass, following which the diameters of the cysts were measured. The volume of the lesion was calculated by approximating the multifocal cyst as a single lumped ellipse, excluding the fatty portion; measuring the width (α), length (β), and height (γ); and applying the formula for the volume of an ellipse (V = 4/3 π abc [mm^3^]; a = 1/2α, b = 1/2β, c = 1/2γ). This is illustrated in Supplementary Fig. 3A and 3B. The endometriotic cysts with the ovaries were fixed with 10% phosphate-buffered formalin, embedded in paraffin, cut into sections of 4 μm thickness, and examined by means of routine immunohistochemical analysis, as described below. Immunohistochemical staining was performed as described previously [16]. For heat-induced epitope retrieval, deparaffinized sections in 0.01 mM citrate buffer were heated for 20 min at 95 °C in a microwave oven. Immunohistochemical staining was performed according to the avidin–biotin immunoperoxidase method using the Histofine® SAB-PO kit (Nichirei, Tokyo, Japan). Endogenous peroxidase activity was blocked by incubation with 0.3% H_2_O_2_ in methanol for 20 min, while non-specific Ig binding was blocked by incubation for 10 min in PBS with 10% normal serum and the corresponding secondary antibody. The sections were incubated at 4 °C overnight with the following primary antibodies against: CD10 (sc-9149, 1:200, Santa Cruz Biotechnology [for human paraffin-embedded tissues]), IL-1β (1:100, Cell Signaling Technology), Ki67 (AB9260, 1:300, Merck KGaA, Darmstadt, Germany), and 4-hydroxynonenal (4-HNE) (BS-6313R, 1:400; Bioss Antibodies, Woburn, MA, USA). The sections were then rinsed and incubated with biotinylated secondary antibodies for 10 min. After washing with PBS, the sections were further incubated with horseradish peroxidase-conjugated streptavidin for 5 min and finally treated with diaminobenzidine in 0.01% H_2_O_2_ for 5 min. The slides were counterstained with Meyer’s hematoxylin, following which the stained sections were observed under a microscope (Axio Imager 2, Zeiss, Oberkochen, Germany).

Stained areas inside the endometrial cyst epithelium were quantitated using ImageJ, threshold 235 (IL-1β)/180 (Ki67). The average values were calculated based on the stained area ratio of randomly selected fields of view for at least two cysts (OE) or magnified images (EM) in each of the four mice, and were compared between two groups. Quantification of 4-HNE-positive ovarian follicles was carried out by measuring the ratio of immunopositive follicles in each follicular area using ImageJ (threshold 165).

### Statistical analyses

Statistical analyses were performed using Student’s *t*-test and one-way analysis of variance with Prism 8 software (GraphPad, San Diego, CA, USA). Differences were considered significant at *P* < 0.05. The data are expressed as mean ± standard error of mean, unless specified otherwise.

## Results

### NLRP3 inflammasomes were upregulated in OE

**Fig. 1:**
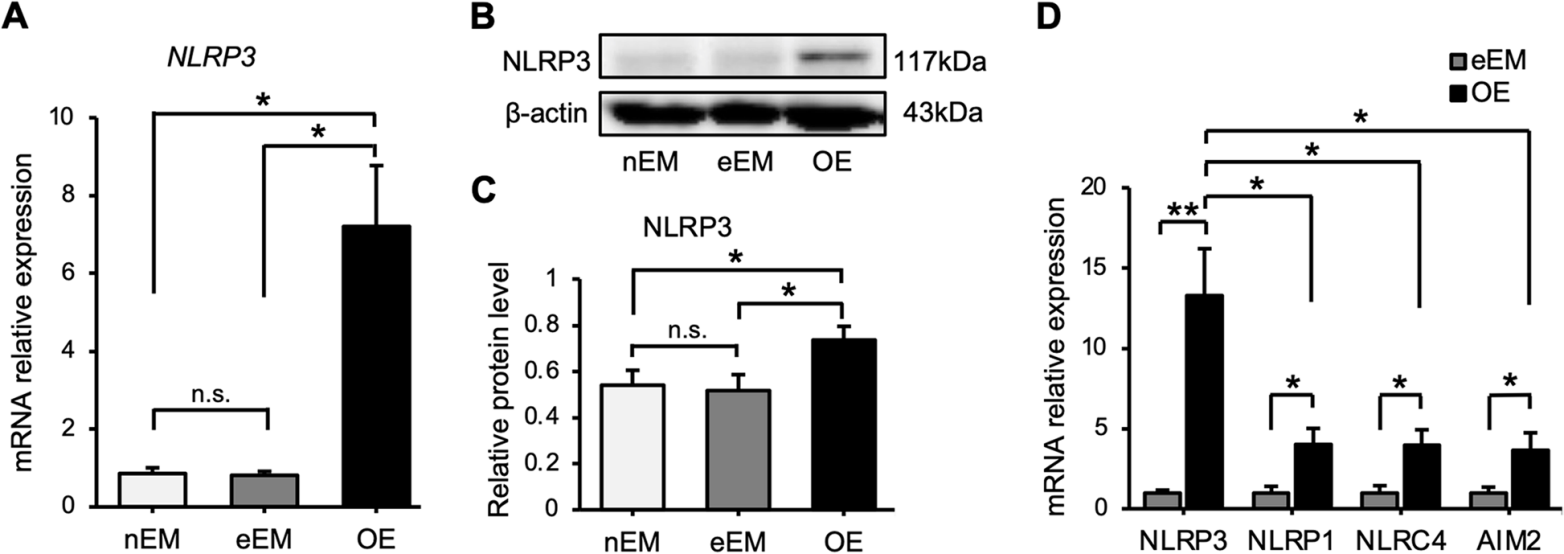
Expression of inflammasomes in n/eEM and OE tissues. (A) Relative mRNA expression level of *NLRP3* was quantified using qRT-PCR. Expression levels are shown relative to *GAPDH*. Data are shown as mean ± SEM of samples obtained from patients and assayed in duplicate; nEM (*n* = 10), eEM (*n* = 12), and OE (*n* = 10). (B) The NLRP3 protein levels in n/eEM and OE tissues were assessed using western blot. β-actin was used as a loading control. These results are representative of three separate experiments. (C) Relative protein levels of NLRP3 were quantified. Protein levels are shown relative to those of β-actin. Data are shown as mean ± SEM of samples obtained from patients; nEM (*n* = 3), eEM (*n* = 4), and OE (*n* = 4). (D) Relative mRNA expression levels of *NLRP3*, *NLRP1*, *NLRC4*, and *AIM2* were quantified. Expression levels are shown relative to those of *GAPDH*. Data are shown as mean ± SEM of assays conducted in duplicate from samples obtained from patients; eEM (*n* = 6) and OE (*n* = 5). Statistical analyses were conducted using one-way ANOVA followed by Dunnett’s multiple comparison test. *P < 0.05; **P < 0.01; n.s., not significant; n/eEM, eutopic endometrium without/with endometriosis, *OE* ovarian endometriosis

To identify whether NLRP3 is involved in endometriosis, we detected the expression of NLRP3 in e/nEM and OE using qRT-PCR and western blot analysis. As shown in Fig. 1A–C, NLRP3 gene expression and protein levels were significantly increased in OE compared with those in e/nEM. There was no significant difference between nEM and eEM; therefore, eEM and OE were selected for subsequent experiments.

Other inflammasomes involved in the caspase1-mediated pathway that activate IL-1β were also upregulated in OE, as compared to EM. However, NLRP3 was more highly expressed in EM than in OE (Fig. 1D).

We then examined whether the same trend was observed in the cells isolated from patients. To determine the origin of these isolated cells, we performed immunocytochemistry for CD10, which is a marker of ESCs (Supplemental Fig. 1A), and stromal cell markers, vimentin and cytokeratin [26–28]. ESCs and CSCs were positive for all markers, but those incubated without a primary antibody did not luminesce at the same fluorescence intensity (Supplemental Fig. 1B).

**Fig. 2:**
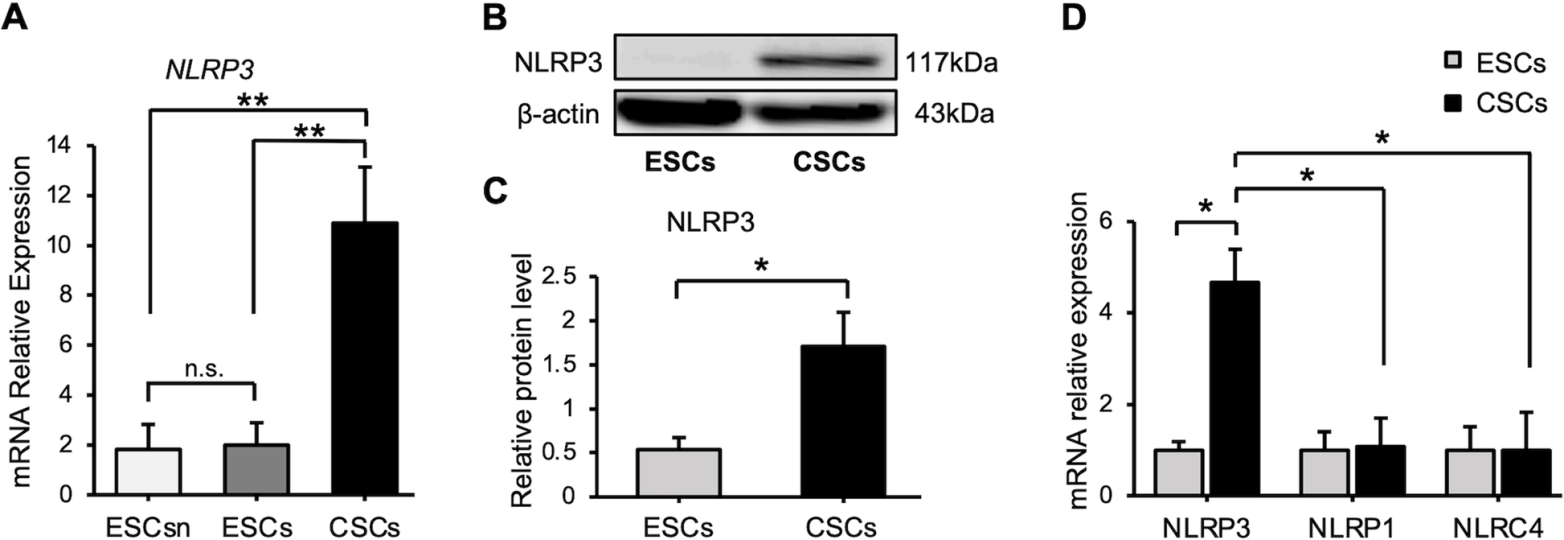
Expression of NLRP3 in ESCs and CSCs. (A) Relative mRNA expression level of *NLRP3* was quantified using qRT-PCR. Expression levels are shown relative to those of *GAPDH*. Data are shown as mean ± SEM of assays conducted in duplicate on samples obtained from patients; ESCsn (*n* = 6), ESCs (*n* = 6), and CSCs (*n* = 6). (B) The protein levels of NLRP3 in ESCs and CSCs were assessed using western blot. β-actin was used as a protein loading control. These results are representative of three independent experiments. (C) The relative protein levels of NLRP3 were quantified. Protein levels are shown relative to those of β-actin in each group. Data are shown as mean ± SEM from patients; ESCs (*n* = 6) and CSCs (*n* = 5). (D) Relative mRNA expression levels of *NLRP3*, *NLRP1*, and *NLRC4* were quantified. Expression levels are shown relative to those of *GAPDH*. Data are shown as mean ± SEM of assays conducted in duplicate on samples obtained from patients; ESCs (*n* = 4) and CSCs (*n* = 5). Statistical significances were calculated using Student’s *t*-test (A and C) and one-way ANOVA, followed by Dunnett’s multiple comparison test (D). *P < 0.05; **P < 0.01; n.s., not significant; ESCsn, eutopic endometrium-derived stromal cells, without endometriosis; ESCs, eutopic endometrium-derived stromal cells, with endometriosis; CSCs, ovarian endometriosis (chocolate cyst)-derived stromal cells

As shown in Fig. 2A, NLRP3 mRNA levels were also significantly higher in CSCs than in ESCsn and ESCs. There was no significant difference between ESCsn and ESCs; therefore, ESCs and CSCs were selected for subsequent experiments. As shown in Fig. 2B–C, NLRP3 protein levels were also significantly higher in CSCs, compared to those in ESCs. In contrast, the expression levels of NLRP1 and NLRC4 were not significantly different between CSCs and ESCs (Fig. 2D).

### MCC950 decreases the viability of CSCs

**Fig. 3:**
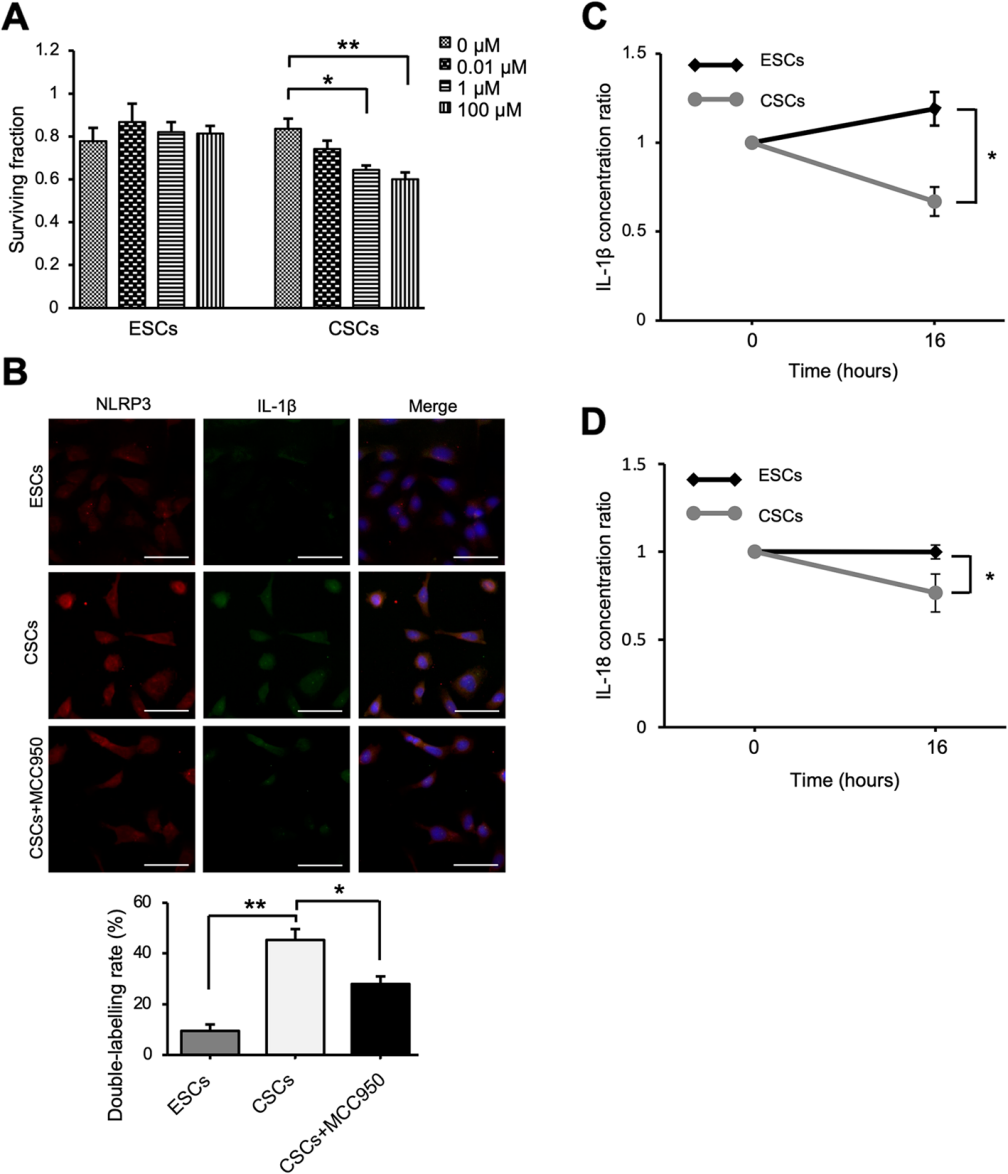
Effects of MCC950 on ESCs and CSCs. (A) ESCs and CSCs were treated with MCC950 (0.01, 1, and 100 μM) and the surviving fraction was measured after a 24 h incubation. Data are shown as mean ± SEM of triplicate samples obtained from patients; ESCs (*n* = 4) and CSCs (*n* = 4). (B) Immunofluorescence analysis of the expression of NLRP3 and IL-1β in ESCs and CSCs. Representative immunostaining images (upper) and quantitative analysis (lower) of the ratio of NLRP3/IL-1β co-labeled cells to total cells after incubating with or without MCC950 (100 μM) for 16 h. Data are shown as mean ± SEM obtained from primary cell cultures; ESCs (*n* = 3) and CSCs (*n* = 3); Scale bar: 50 μm. Ratio of IL-1β (C) and IL-18 (D) levels in CSC culture supernatant from untreated and MCC950-treated (100 μM) cultures after 16 h. Data are shown as mean ± SEM of duplicates obtained from primary cell cultures; (C) ESCs (*n* = 7) and CSCs (*n* = 8), (D) ESCs (*n* = 7) and CSCs (*n* = 9). Statistical significances were calculated using one-way ANOVA followed by Dunnett’s multiple comparison test (A and B) and Student’s *t*-test (C and D). *P < 0.05 and **P < 0.01; *ESCs* eutopic endometrium-derived stromal cells, with endometriosis, *CSCs* ovarian endometriosis (chocolate cyst)-derived stromal cells

Because there was an increase in the expression of NLRP3 in CSCs, MCC950 was added to the cultured cells, to evaluate the effect of inhibition of IL-1β. When ESCs and CSCs were treated with 0.01, 1, and 100 μM MCC950, the survival fraction of CSCs at 24 h was 89 ± 4.1%, 78 ± 3.4%, and 73 ± 5.8%, respectively, compared to that of CSCs treated with 0 μM MCC950 (*P* = 0.14, 0.011, and 0.0065, respectively), whereas the viability of ESCs did not change significantly (Fig. 3A).

### Attenuation of IL-1β secretion upon MCC950 pre-treatment in CSCs

The effect of MCC950 on ESCs and CSCs was evaluated. Immunofluorescence experiments indicated that the number of NLRP3 and IL-1β double-labeled cells in CSCs was higher than that in ESCs. Furthermore, when CSCs were treated with MCC950, the expression of IL-1β was downregulated, and the number of double-labeled cells was reduced (Fig. 3B).

Next, IL-1β secretion in the cell supernatant was examined, and we found that the concentration of IL-1β was higher in the supernatant of CSCs than in that of ESCs, although this difference was not statistically significant. The level of IL-1β in the CSC supernatant was significantly reduced after 16 h of treatment with MCC950 (Fig. 3C). Since both IL1β and IL-18 are activated in the NLRP3-caspase1 pathway [19], IL-18 was also evaluated, and the results were similar (Fig. 3D). In addition, when we measured the expression of proteins in cell lysates with/without MCC950, there was a significant reduction in the levels of caspase-1 in CSCs after the addition of MCC950, as compared to that in ESCs (Supplemental Fig. 2A–B).

### MCC950 prevents progression of OE cysts in murine models

**Fig. 4:**
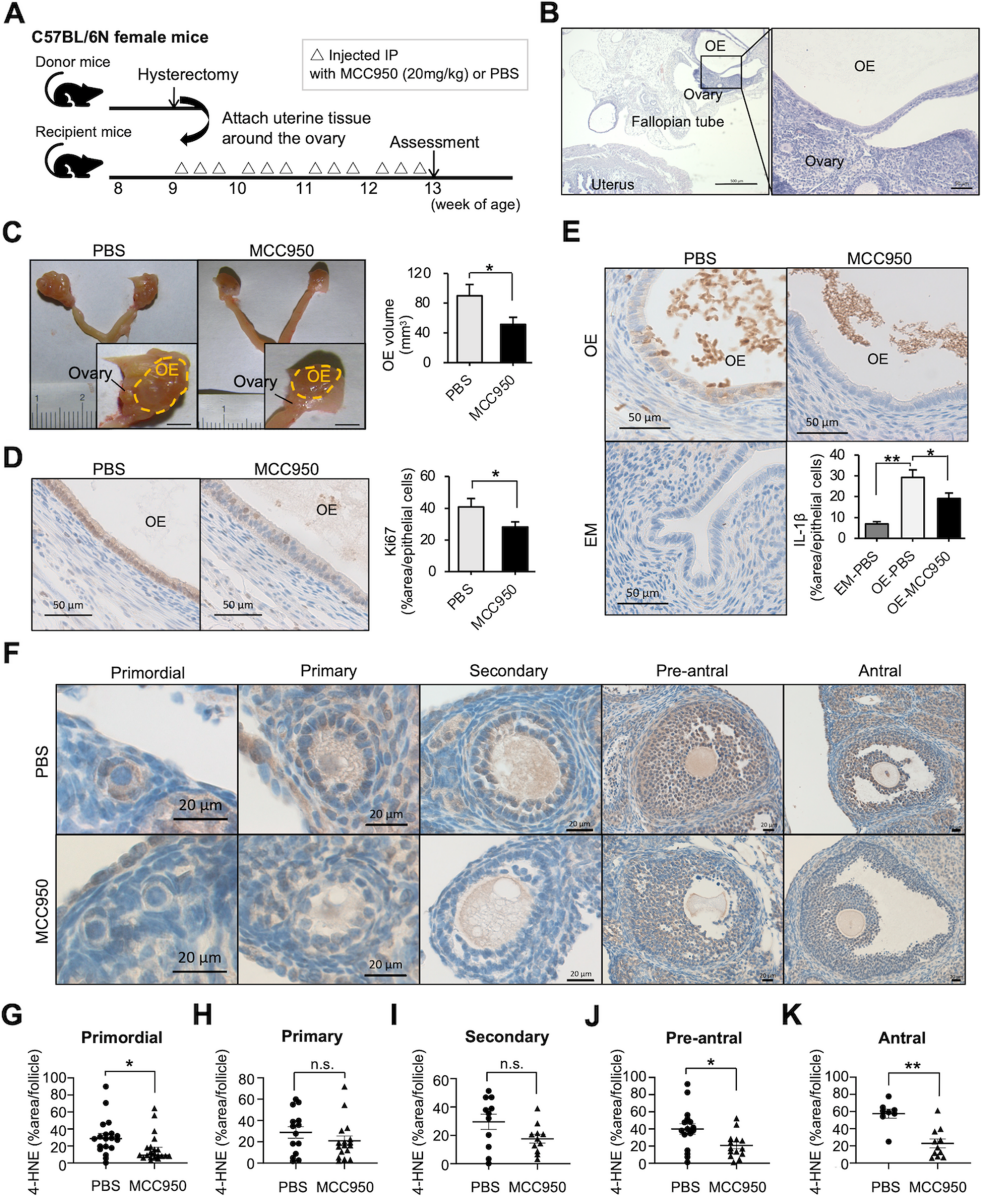
Effects of MCC950 on endometriosis lesions and ovaries of a murine endometriosis model. (A) Experimental design of the role of MCC950 in a murine ovarian endometriosis model. (B) Hematoxylin and eosin staining of lesions; Scale bar: 500 μm (left) and 50 μm (right). (C) Macroscopic view of lesions (left) and volumes of lesions per ovary were assessed (right). Data are presented as mean ± SEM; PBS-treated (*n* = 8 mice, 16 ovaries) and MCC950-treated (*n* = 7 mice, 14 ovaries); scale bar: 2 mm. (D and E) Histological analysis of murine endometriosis lesions treated with vehicle and MCC950, by staining for Ki67 (D) and IL-1β (E). Representative immunohistochemical localization (left, scale bar: 50 μm), and quantitative analysis of positive epithelial area ratio of the endometriotic cyst wall or eutopic endometrium (right). (F) Representative 4-HNE immunohistochemical results of ovarian follicles in primordial, primary, secondary, pre-antral, and antral stages; scale bar: 20 μm. (G–K) Quantitative analysis of each follicular stage of the ovary. Data are shown as mean ± SEM of at least eight follicles from four mice. Statistical significances were calculated using Student’s *t*-test (C, D, and G–K) and one-way ANOVA followed by Dunnett’s multiple comparison test (E). *P < 0.05; **P < 0.01; n.s., not significant; *IP* intraperitoneal, *OE* ovarian endometriosis, *EM* eutopic endometrium, *4-HNE* 4-hydroxynonenal

The experimental protocol using the murine OE model is summarized in Fig. 4A. Both the MCC950-treated and PBS-treated groups were euthanized four weeks after the operation (at the age of 13 weeks), following which the endometriotic lesions were evaluated. Single or multiple cystic lesions were recognized in association with the bilateral ovaries (Fig. 4B). After treatment with MCC950 for four weeks following implantation with minced murine uterine tissues, the volume of lesions was significantly reduced compared to that in the PBS-treated group (89 ± 15 *vs.* 49 ± 9.3 mm^3^ per ovary, *P* < 0.05; Fig. 4C, Supplementary Fig. 3C). To evaluate the proliferative activity of the endometriotic lesions, the ratio of Ki67-positive cells was calculated. The number of Ki67-positive epithelial cells in the endometriotic lesions decreased significantly after MCC950 treatment (40.8 ± 5.3% *vs.* 28.1 ± 3.2%, *P* < 0.05; Fig. 4D).

We then evaluated the effect of MCC950 on IL-1β expression in murine endometriotic cysts. The levels of IL-1β in endometriotic cysts after MCC950 treatment were assessed by means of immunohistochemical analysis. The IL-1β-positive area in the epithelial cells was higher in the OE cysts than in the EM of the same animals after PBS treatment, which significantly decreased after MCC950 treatment (OE-PBS [29.3 ± 3.6%] *vs.* OE-MCC950 [19.1 ± 2.6%], *P* < 0.05; Fig. 4E).

### MCC950 reduced endometriosis-induced oxidative stress in granulosa cells of the murine model

We evaluated oxidative stress in granulosa cells of ovarian follicles, as it has been reported that iron and oxidative stress are major factors in the impaired fertility observed in OE and that there is a significant increase in the level of the oxidative stress marker 4-HNE in the primordial, preantral, and antral follicles of the murine model of OE [25]. 4-HNE is one of the most specific lipid peroxidation products in the iron-catalyzed Fenton reaction. Representative data of the different maturation stages of 4-HNE-stained follicles are summarized in Fig. 4F. In the primordial, pre-antral, and antral follicles, 4-HNE levels in the granulosa cells were lower in the MCC950-treated group than in the PBS-treated group (Fig. 4G–K). In addition, the number of follicles was higher in the MCC950-treated group (Supplemental Fig. 4).

## Discussion

In this study, we investigated the NLRP3 inflammasome, which contributes to IL-1β activation, as a target for the non-hormonal treatment of OE. The involvement of IL-1β in endometriosis has been studied [17, 29, 30], and research on the treatment of endometriosis using IL-1β receptor antibodies has been reported [31]. However, among patients who received a monoclonal antibody against IL-1β, adverse events, such as infection, are frequently reported [32]. Inflammasomes, which consist of NLR, the adaptor protein apoptosis-associated speck-like protein containing CARD, and the effector molecule pro-caspase-1, facilitate the cleavage and activation of caspase-1, which leads to the maturation of IL-1β [33]. The NLR family of proteins is a group of pattern-recognition receptors (PRRs) [34], and some PRRs assemble the inflammasome complex after sensing their respective stimuli. The NLRP3 inflammasome is activated by various pathogen-derived ligands and physiological aberrations, resulting in DAMPs. The NLRP1 inflammasome senses *Bacillus anthracis* toxin, and the pathogen-associated proteins released by pathogenic bacteria cause NLRC4 to assemble the inflammasome complex. DNA viruses and intracellular bacteria release DNA during infection, which activates the AIM2 inflammasome [35].

In the present study, while NLRP3 expression levels were elevated in cultured CSCs, as compared to those in ESCs, there were no differences in NLRP1 and NLRC4 levels, indicating that NLRP3 might be involved in the pathogenesis of endometriosis. We detected notably increased NLRP3 levels in the OE tissues from surgical specimens, as compared to those in the EM, which is consistent with other studies [36]. Additionally, our results suggest that the EM is similar with and without endometriosis, and that NLRP3 is elevated due to inflammation during the OE process and due to the contents of the OE itself. Therefore, NLRP3 inhibitors do not affect the EM and may be useful in the treatment of OE in patients undergoing fertility treatment. In contrast, the expression of other NLRs was upregulated in OE compared to that in the EM, although to a lesser extent than that of NLRP3. Considering the heterogeneity in clinical conditions, it is possible that other NLRs are also elevated in OE cysts in response to stimuli, such as infection of the cyst or bacteria in the abdominal cavity. NLRP3, which is also highly expressed in cultured cells, is continuously activated in clinical conditions due to continued exposure to the contents of endometrial cysts, which can progress to become DAMPs. In this regard, NLRP3 is more highly activated in the stressful environment of exposure to the contents of endometrial cysts than in cultured cells, and the therapeutic strategy of inhibiting NLRP3 may be more effective in vivo than in vitro. Additionally, MCC950 does not block the major antimicrobial inflammasomes, NLRC4 and NLRP1 [21]. The NLRP3 level was significantly higher than that of other NLRs in OE samples and CSCs; therefore, the specific inhibition of NLRP3 by MCC950 could suppress IL-1β production in endometriosis, while essential responses against bacterial infections may remain intact.

In previous reports, several peritoneal models have been reported as murine models of endometriosis [37–39], and in this study, we used an OE model that was recently established [25]. Therefore, we assessed the effects on the ovaries, especially the follicles. With respect to the effects of MCC950 on the ovary, a previous report suggested that administration of MCC950 inhibits ovarian aging and improves fertility in mice [40]. The association of increased oxidative stress in follicles with decreased fertility has been reported in mouse models of endometriosis [25]. Our results indicate that administration of MCC950 reduced the oxidative stress of granulosa cells in follicles and further increased the number of small follicles and antral follicles (Supplemental Fig. 4). Taken together, these effects of MCC950 may improve fertility in murine models and possibly in patients with OE.

The limiting factor of this study is that there are variabilities in OE samples; consequently, there may be individual differences in the effectiveness of NLRP3 inhibition. NLRP3 has been reported to vary with the menstrual cycle [41]; however, this has not been fully investigated. Additionally, MCC950 has not been used in clinical settings because it was found to elevate serum liver enzyme levels in clinical trials for rheumatoid arthritis [42]. Next-generation NLRP3 pathway inhibitors have been developed in clinical trials [43]. Tranilast, an NLRP3 inhibitor that is clinically applied as an anti-allergic drug but is less specific than MCC950 [24, 44], is of interest to us because it may have a positive impact on the treatment of endometriosis.

## Conclusions

The expression of NLRP3 was upregulated in OE samples, as compared to that in EM. Treatment of CSCs with MCC950 suppressed IL-1β production and cell proliferation. The administration of MCC950 reduced endometriotic lesions in a murine model of OE and improved the function of ovaries with endometriosis, suggesting that MCC950 is a potential non-hormonal treatment for endometriosis.

## Supplementary Information

**Additional file 1.** **Supplemental Figure 1.** Identification of primary ESCs and CSCs. (A) Immunohistochemical staining of CD10. eEM of hysterectomized uterus (left) and OE of adnexal resected ovary (right). The ESCs were positive for CD10, while the cortex, medulla, and epithelial cells were negative for it. Scale bar: 200 μm. (B) Immunocytochemical staining of each marker protein. All images are merged images. DAPI images were taken at the same exposure time as CD10, without the inclusion of primary antibodies. DAPI (upper), green; CD10 (middle), green; vimentin (lower), red; fibronectin (lower); scale bar: 100 μm. eEM, eutopic endometrium with endometriosis; OE, ovarian endometriosis; ESCs, eutopic endometrium-derived stromal cells, with endometriosis; CSCs, ovarian endometriosis (chocolate cyst)-derived stromal cells

**Additional file 2.** **Supplemental Figure 2.** Effects of MCC950 on ESCs and CSCs. (A) The protein level of caspase-1 in ESCs and CSCs with/without MCC950 (100 μM), after 12 h of incubation, was assessed using western blot. β-actin was used as a protein loading control. The results are representative. (B) Relative protein levels of caspase-1 were quantified. The ratio of caspase-1 protein levels in the untreated and MCC950-treated (100 μM) cell lysates incubated for 12 h. Data are shown as mean ± SEM from patients; ESCs (*n*=3) and CSCs (*n*=4). Statistical significance was calculated using the Student’s t-test. *P<0.05; ESCs, eutopic endometrium-derived stromal cells with endometriosis; CSCs, ovarian endometriosis (chocolate cyst)-derived stromal cells

**Additional file 3.** **Supplemental Figure 3.** Evaluation of the effects of MCC950 on endometriotic lesions in a murine endometriosis model. (A) The volume of the lesion was calculated by approximating the multifocal cyst as a single lumped ellipse, excluding the fatty portion, and measuring the width (α), length (β), and height (γ). (B) Applying the formula for the volume of an ellipse (V = 4/3 π abc [mm^3^]; a=1/2α, b=1/2β, and c=1/2γ). (C) Representative macrographs of the uterus and ovaries of untreated 13-week-old mouse (control) and murine models with OE lesions treated with PBS or MCC950 (OE + PBS/OE + MCC 950). OE; ovarian endometriosis

**Additional file 4.** **Supplemental Figure 4.** MCC950 improves follicle number in a murine endometriosis model. (A) Representative micrographs of ovarian sections from the PBS and MCC950 groups. (B) Quantification of number of ovarian follicles. Data are presented as mean ± SEM; PBS-treated (*n*=4) and MCC950-treated (*n*=4). Statistical significance was calculated using Student’s t-test. *P<0.05, PMF, primordial follicle; PF, primary follicle; SF, secondary follicle; AF, antral follicle; Scale bar: 200 μm

## Data Availability

The datasets used and analyzed during the current study are available from the corresponding author upon reasonable request.

## Abbreviations

**OE**: Ovarian endometriosis
**NLRP**: Nucleotide-binding oligomerization domain, leucine rich repeat, and pyrin domain
**IL-1β**: Interleukin-1 beta
**EM**: Eutopic endometrium
**CSCs**: Primary human endometriotic cyst-derived stromal cells
**ESCs**: Primary human endometrial stromal cells, obtained from patient with endometriosis
**ESCsn**: Primary human endometrial stromal cells, obtained from patient without endometriosis
**PBS**: Phosphate-buffered saline
**GnRH**: Gonadotropin releasing hormone
**NLR**: Nucleotide-binding oligomerization domain-like receptor
**NLRC**: NLR family caspase recruitment domain (CARD)-containing protein
**CIS**: Carcinoma in situ
**eEM**: Endometrial tissues from patients with endometriosis
**nEM**: Endometrial tissues from patients without endometriosis
**qRT-PCR**: Quantitative reverse transcription-polymerase chain reaction
**DMEM**: Dulbecco’s modified Eagle’s medium
**AIM2**: Absent in melanoma 2
**GAPDH**: Glyceraldehyde-3-phosphate dehydrogenase
**PBST**: Phosphate-buffered saline with Tween
**SFM**: Serum-free media
**ELISA**: Enzyme-linked immunosorbent assay
**4-HNE**: 4-Hydroxynonenal
**ANOVA**: Analysis of variance
**SEM**: Standard error of mean
**PRPs**: Pattern recognition receptors
**DAMP**: Danger-associated molecular pattern
