# Supplementary figures and images for: Effectiveness of NLRP3 Inhibitor as a Non-Hormonal Treatment for ovarian endometriosis
Source: Reprod Biol Endocrinol. 2022 Mar 29;20:58. doi: 10.1186/s12958-022-00924-3 (PMC8966161; doi:10.1186/s12958-022-00924-3)

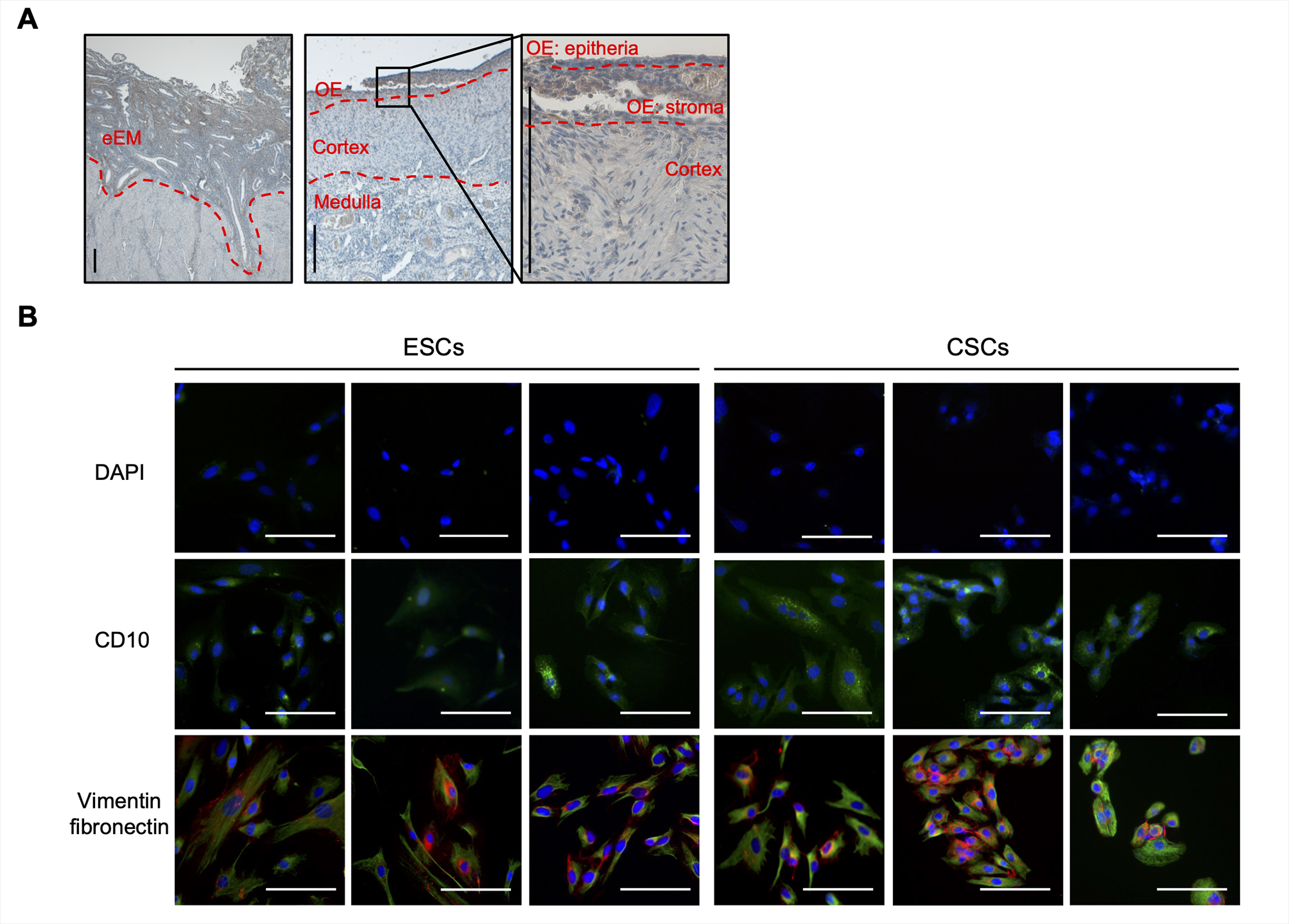

Supplement: Supplementary file 1 — Additional file 1. Supplemental Figure 1. Identification of primary ESCs and CSCs. (A) Immunohistochemical staining of CD10. eEM of hysterectomized uterus (left) and OE of adnexal resected ovary (right). The ESCs were positive for CD10, while the cortex, medulla, and epithelial cells were negative for it. Scale bar: 200 μm. (B) Immunocytochemical staining of each marker protein. All images are merged images. DAPI images were taken at the same exposure time as CD10, without the inclusion of primary antibodies. DAPI (upper), green; CD10 (middle), green; vimentin (lower), red; fibronectin (lower); scale bar: 100 μm. eEM, eutopic endometrium with endometriosis; OE, ovarian endometriosis; ESCs, eutopic endometrium-derived stromal cells, with endometriosis; CSCs, ovarian endometriosis (chocolate cyst)-derived stromal cells [file 12958_2022_924_MOESM1_ESM.tif]

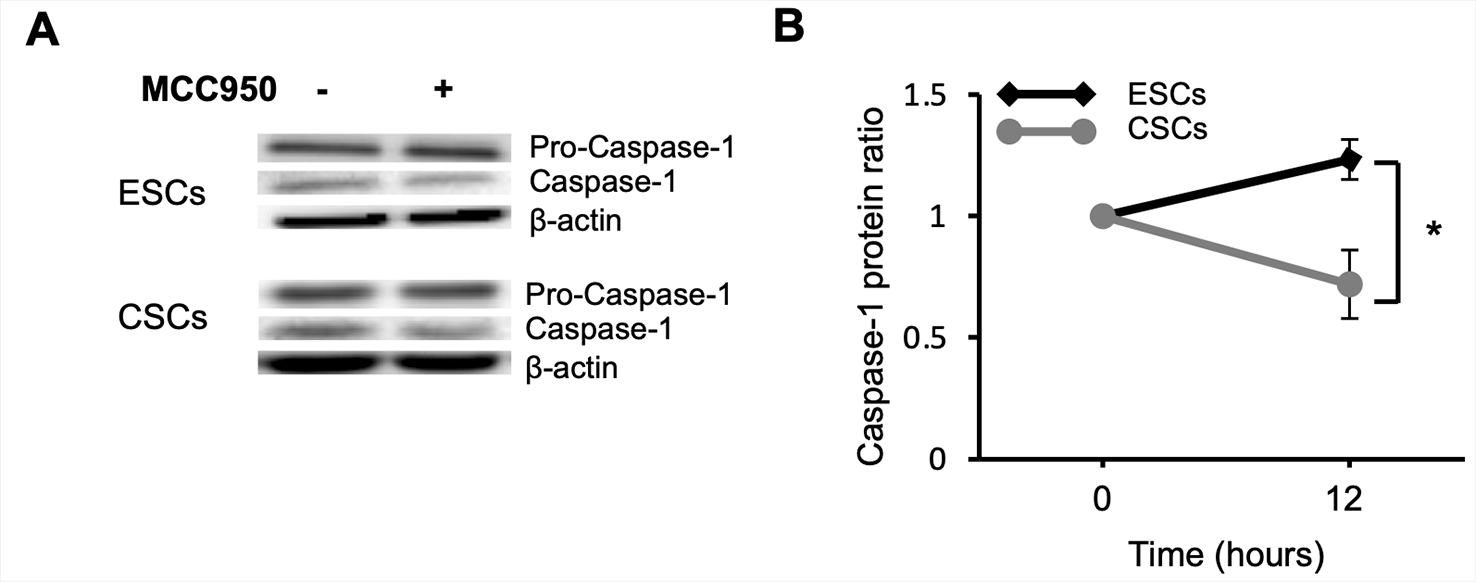

Supplement: Supplementary file 2 — Additional file 2. Supplemental Figure 2. Effects of MCC950 on ESCs and CSCs. (A) The protein level of caspase-1 in ESCs and CSCs with/without MCC950 (100 μM), after 12 h of incubation, was assessed using western blot. β-actin was used as a protein loading control. The results are representative. (B) Relative protein levels of caspase-1 were quantified. The ratio of caspase-1 protein levels in the untreated and MCC950-treated (100 μM) cell lysates incubated for 12 h. Data are shown as mean ± SEM from patients; ESCs (n=3) and CSCs (n=4). Statistical significance was calculated using the Student’s t-test. *P<0.05; ESCs, eutopic endometrium-derived stromal cells with endometriosis; CSCs, ovarian endometriosis (chocolate cyst)-derived stromal cells [file 12958_2022_924_MOESM2_ESM.tif]

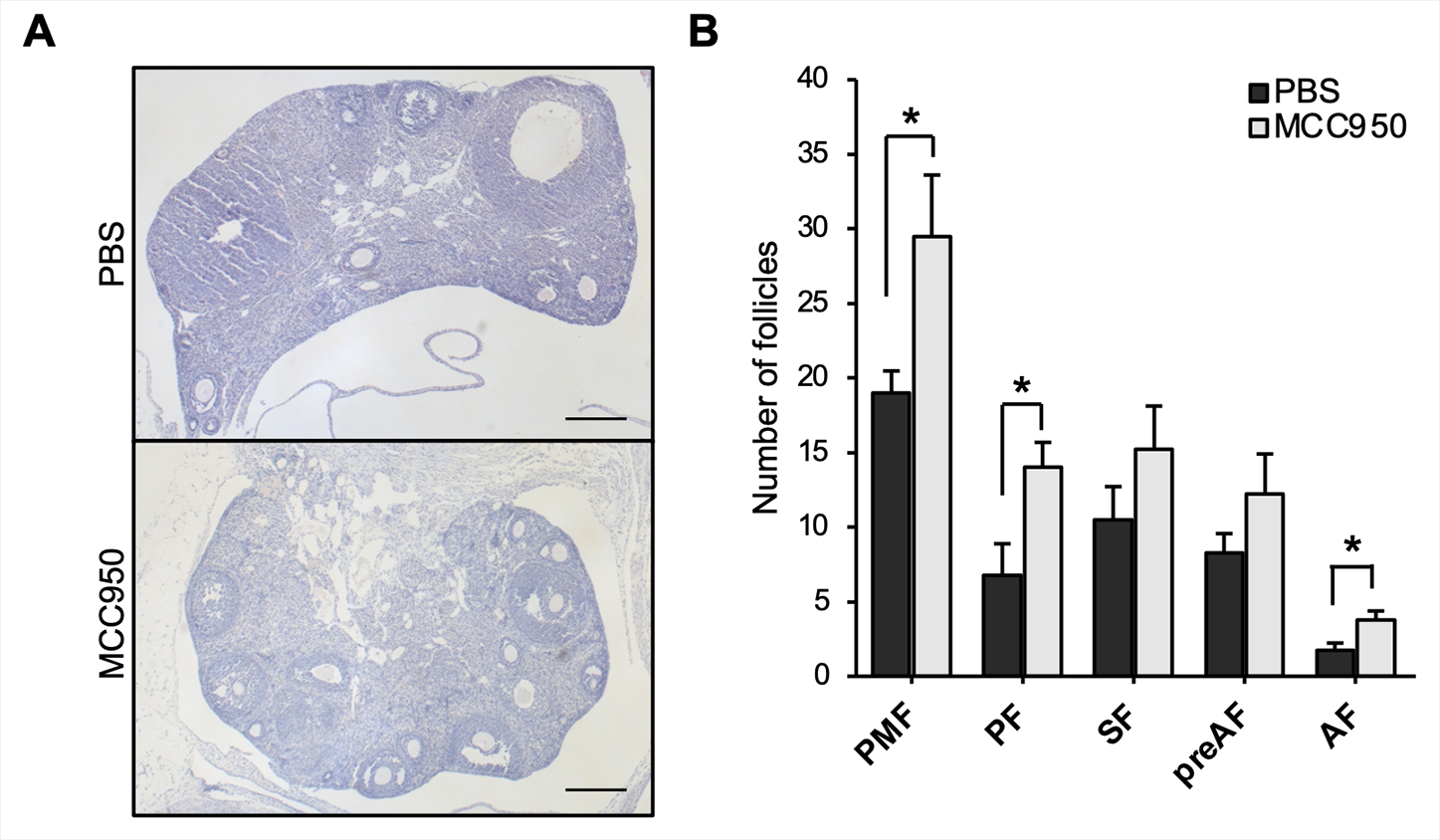

Supplement: Supplementary file 3 — Additional file 3. Supplemental Figure 3. Evaluation of the effects of MCC950 on endometriotic lesions in a murine endometriosis model. (A) The volume of the lesion was calculated by approximating the multifocal cyst as a single lumped ellipse, excluding the fatty portion, and measuring the width (α), length (β), and height (γ). (B) Applying the formula for the volume of an ellipse (V = 4/3 π abc [mm3]; a=1/2α, b=1/2β, and c=1/2γ). (C) Representative macrographs of the uterus and ovaries of untreated 13-week-old mouse (control) and murine models with OE lesions treated with PBS or MCC950 (OE + PBS/OE + MCC 950). OE; ovarian endometriosis [file 12958_2022_924_MOESM3_ESM.tif]

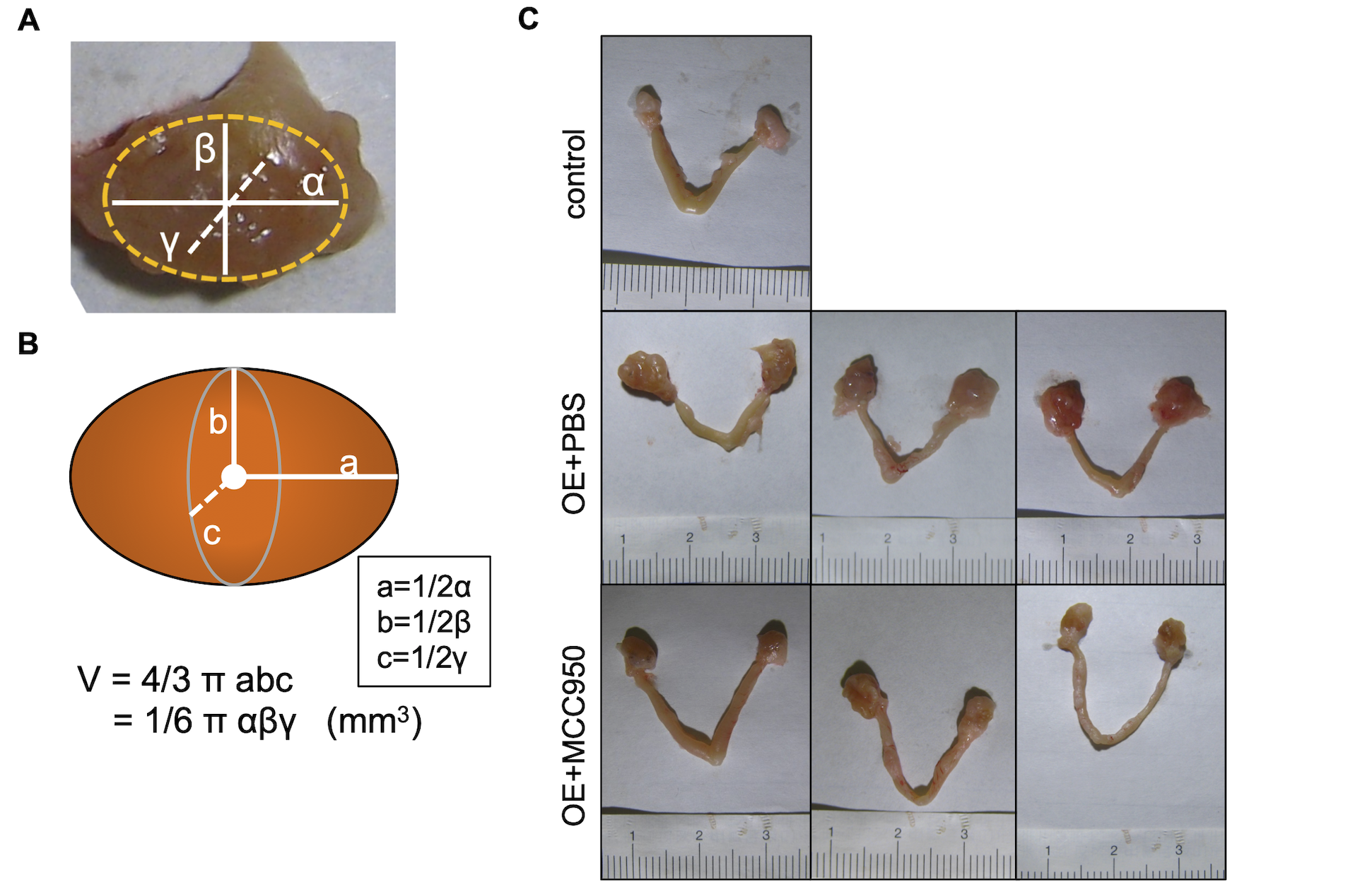

Supplement: Supplementary file 4 — Additional file 4. Supplemental Figure 4. MCC950 improves follicle number in a murine endometriosis model. (A) Representative micrographs of ovarian sections from the PBS and MCC950 groups. (B) Quantification of number of ovarian follicles. Data are presented as mean ± SEM; PBS-treated (n=4) and MCC950-treated (n=4). Statistical significance was calculated using Student’s t-test. *P<0.05, PMF, primordial follicle; PF, primary follicle; SF, secondary follicle; AF, antral follicle; Scale bar: 200 μm [file 12958_2022_924_MOESM4_ESM.tiff]
